# Development of a relative molar sensitivity–based HPLC method for quantifying acetaminophen, two beta-lactams, amiodarone, and its metabolite

**DOI:** 10.1186/s40780-026-00615-w

**Published:** 2026-08-03

**Authors:** Takashi Ohtsuki, Yume Enomoto, Akane Yasuda, Risa Imai, Nonoka Seto, Makoto Hashimoto, Miyuki Matsushita, Satoru Morikawa, Hiroshi Matsufuji

**Affiliations:** 1 College of Bioresource Sciences, Nihon University, 1866 Kameino, Fujisawa, Kanagawa Japan; 2 Hitachi High-Tech Analysis Corporation, 1-17-1, Toranomon, Minato-ku, Tokyo Japan

**Keywords:** Relative molar sensitivity, HPLC, High-performance liquid chromatography, Acetaminophen, Cefmetazole, Cefazolin, Amiodarone, *N*-desethylamiodarone, Therapeutic drug monitoring

## Abstract

**Background:**

Therapeutic drug monitoring (TDM) is essential for drugs with narrow therapeutic ranges and high pharmacokinetic variability. However, significant barriers hinder TDM implementation. Outsourcing of testing can delay timely clinical decision-making, with possible delays of up to 1 week. Additionally, management of analyte-specific certified reference material (CRM) presents substantial operational challenges, particularly for unstable compounds. The present study therefore developed and validated a high-performance liquid chromatography (HPLC) method based on relative molar sensitivity (RMS) of stable non-analyte reference materials (carbamazepine and caffeine) to quantify five clinically important analytes—acetaminophen, two beta-lactam antibiotics (cefmetazole and cefazolin), and amiodarone and its active metabolite, *N*-desethylamiodarone.

**Methods:**

RMS values were calculated as the ratio of the analyte and non-analyte reference material calibration curve slopes. CRM solutions were used at 6 to 10 concentrations per analyte. HPLC analysis utilized analyte-specific detection wavelengths in the range 240–275 nm. For validation, control serum samples were spiked at 2 or 3 concentrations per analyte and processed via spin-column solid-phase extraction. Quantitative accuracy was assessed by comparing the RMS-based results with conventional absolute calibration methods.

**Results:**

All calibration curves demonstrated excellent linearity, with correlation coefficients in the range 0.9993–1.0000. Calibration curve slope reproducibility was high, with relative standard deviations of 0.3%–1.5% (*n* = 3). Quantitative analyses of spiked serum samples showed close agreement between the RMS and conventional methods, with relative differences not exceeding ± 0.50% for either calibrant for any analyte. Both methods achieved comparable precision, with relative standard deviations of < 4.6%.

**Conclusions:**

The validated RMS-based HPLC method provides accurate and precise quantification of structurally diverse drugs using stable, the International System of Units (SI)-traceable, non-analyte reference materials. This approach eliminates the need for analyte-specific authentic standards for routine analyses, thus substantially reducing procurement costs and addressing the instability of some analytes. The rapid and accurate analysis enables timely reporting, potentially leading to improved clinical outcomes through expedited in-house TDM implementation in hospital laboratories with limited resources.

## Background

Insufficient exposure of target tissues to drugs generally leads to failure to achieve the desired therapeutic effect, whereas excessive drug concentrations in the blood increase the risk of adverse events and toxicity. Drug doses are thus adjusted with reference to the therapeutic concentration range. However, both therapeutic response and toxicity are typically continuous phenomena, such that responses do not necessarily change abruptly at the upper and lower limits of the therapeutic range. Consequently, establishing appropriate dosing regimens is particularly challenging for drugs with narrow therapeutic ranges.

Therapeutic drug monitoring (TDM) is a clinical practice that ensures efficacy and improves safety by monitoring drug concentrations in the blood in order to individualize the dosing regimen (including dose and dosing interval) [1, 2]. Drugs for which TDM is beneficial typically have: (i) predictable dose–concentration relationships, (ii) narrow therapeutic ranges, (iii) substantial interindividual pharmacokinetic variability, and (iv) a risk of serious concentration-dependent adverse events [1, 2]. For such drugs, it is necessary to establish an optimal patient-specific dosing regimen by adjusting the dose based on the drug’s concentration in the blood [3].

In order to implement TDM, many healthcare facilities must outsource drug concentration analyses to commercial reference laboratories. However, up to 1 week may be required to obtain results from an external laboratory [4, 5], which poses challenges for cases in which timely therapeutic decision-making is necessary [6]. Therefore, analytical methods capable of providing rapid, high-precision measurements of blood drug concentrations within healthcare facilities are urgently needed.

Recent advances in instrumentation development have yielded practical, high-performance liquid chromatography (HPLC) systems specifically designed for rapid routine clinical analyses without the need for specialized expertise [7–9]. Despite these advances, however, the complexity of reference standard management practices remains a significant barrier to routine implementation [10]. Although HPLC instrumentation has become increasingly accessible, the management of multiple certified reference materials (CRMs) for different analytes requires substantial ongoing operational resources for procurement of analyte-specific standards, maintenance of precise storage conditions (e.g., temperature, humidity, and light protection), periodic stability testing, and regular replacement due to degradation. Additionally, the cost of procuring CRMs with clearly assigned purity values—essential for metrological traceability—can be significant. This multifaceted operational burden imposes direct costs (e.g., material procurement and storage infrastructure), indirect labor costs (e.g., handling and testing), and opportunity costs that present a formidable obstacle for hospital laboratories seeking cost-effective in-house TDM implementation.

The above-mentioned operational challenges are further compounded by the limited availability of CRMs for many analytes. The use of conventional external (absolute) calibration methods in quantitative HPLC analysis generally requires authentic analyte reference standards to construct calibration curves for accurate results [11]. However, CRMs may not be available for all analytes. Although the Code d’Indexation des Matériaux de Référence international reference material database includes ~ 10,000 reference materials [12, 13], it does not comprehensively cover the diverse range of analytes encountered in practical clinical applications. Various quantitative analytical HPLC methods based on relative molar sensitivity (RMS) have been developed as a means of overcoming the limitations in CRM availability [14]. RMS is a dimensionless coefficient that represents the ratio of the detector response (per molar unit) of the analyte to that of a different reference substance under identical chromatographic conditions. If the precise RMS is known, the analyte can be quantified using the reference substance without employing an analyte reference standard [15–18]. Therefore, this approach can be used to address problems associated with reference standard availability and related operational costs. Furthermore, the use of CRMs with guaranteed purity as reference substances can lead to improved reliability of quantitative analyses. Although immunoassays offer rapid turnaround times for analyses of selected drugs, limitations include cross-reactivity issues, lack of metabolite specificity, and restricted analyte coverage [19–21]. The RMS-based HPLC method described here addresses these limitations while maintaining analytical rigor and also enables the simultaneous quantification of multiple drugs and their metabolites. In a previous study, we established an RMS-based HPLC method for nine pharmaceutical compounds [22].

The present study focused on therapeutically critical drugs that present specific TDM challenges in order to further demonstrate the clinical utility and versatility of the RMS-based HPLC approach. Acetaminophen carries a risk of hepatotoxicity in overdose situations, necessitating rapid plasma monitoring for timely antidote administration [23]. Beta-lactam antibiotics (e.g., cefmetazole and cefazolin) can exhibit substantial pharmacokinetic variability in critically ill patients, potentially resulting in both subtherapeutic exposure and concentration-related adverse effects (notably neurotoxicity) in high-risk populations, which necessitates TDM for individualized dosing [24–27]. The terminal elimination half-life of amiodarone is prolonged and highly variable (ranging from weeks to months), and its active metabolite, *N*-desethylamiodarone, also has a long half-life [28, 29]. Due to tissue accumulation, amiodarone-related adverse effects and drug interactions may persist after discontinuation of treatment, with toxicity risk being related to cumulative exposure, thus necessitating careful monitoring to balance therapeutic efficacy against cumulative toxicity [28, 30].

**Fig. 1:**
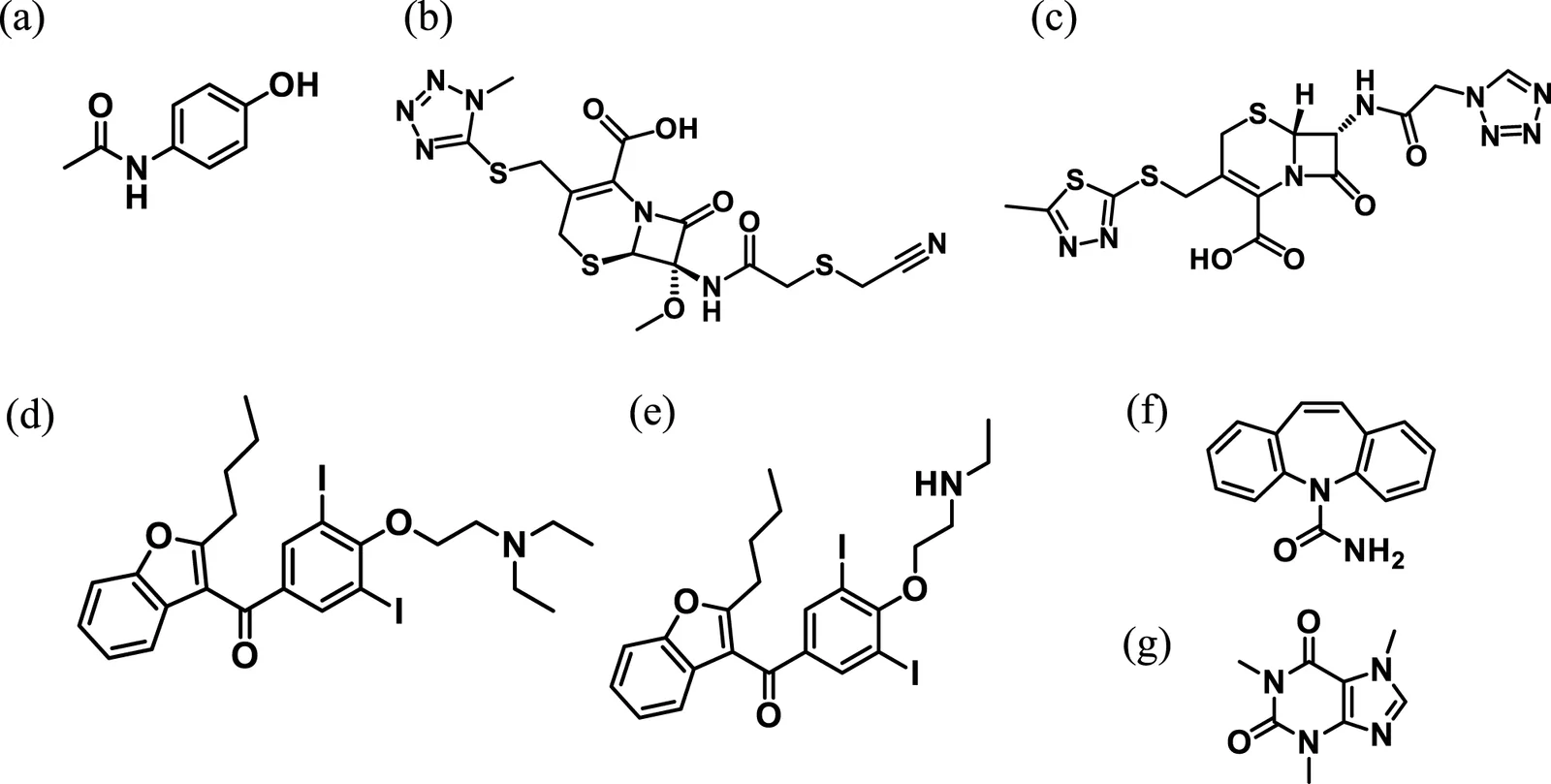
Chemical structures. Chemical structures of the five analytes and two non-analyte reference materials: (**a**) acetaminophen, (**b**) cefmetazole, (**c**) cefazolin, (**d**) *N*-desethylamiodarone, (**e**) amiodarone, (**f**) carbamazepine, and (**g**) caffeine

In this study, we built upon our previous RMS-based HPLC method for nine pharmaceutical compounds [22] to develop and validate quantification methods for five additional analytes: acetaminophen, cefmetazole, cefazolin, amiodarone, and *N*-desethylamiodarone, the active metabolite of amiodarone (Fig. 1). In addition, the applicability of the methods to biological matrices was evaluated using control serum samples spiked with each analyte. The aim of this study was to demonstrate that the RMS-based HPLC method is useful for the accurate, cost-effective quantification of a diverse range of drugs subjected to TDM. The demonstrated utility of the method could facilitate routine in-house analyses of these drugs, thereby improving clinical decision-making by reduced analytical turnaround times.

## Methods

### Samples and reagents

CRM solutions of acetaminophen (300 µg/mL), cefazolin (50 µg/mL), and a mixed CRM solution of amiodarone and *N*-desethylamiodarone (5.0 µg/mL each) were obtained from Hitachi High-Tech Analysis Corporation (Tokyo, Japan). A standard solution of cefmetazole (100 µg/mL) was obtained from Hitachi High-Tech Analysis Corporation (Tokyo, Japan). This concentration was determined by quantitative proton nuclear magnetic resonance spectroscopy (¹H-qNMR) using accurate quantitative NMR with an internal reference substance (AQARI) for absolute quantification. When appropriately certified reference materials are employed in this method, the measurement results are metrologically traceable to SI [31]. All other analytical reagents were of special grade. Control serum samples were prepared by spiking pooled human liquid control serum I (Fujifilm Wako Pure Chemical Industries, Ltd., Osaka, Japan) with each CRM. Spiked samples were aliquoted and stored until analysis at − 30 °C for the other analytes in order to minimize the number of freeze–thaw cycles. Frozen spiked samples were thawed at 25 °C immediately before analysis.

### Instrumentation

Calibration standard solutions of each analyte for HPLC analysis were prepared using a semimicro balance (AUW220D; Shimadzu Corp., Kyoto, Japan). Analytical HPLC was performed using a system equipped with an ultraviolet (UV) absorption detector (LM1010 evaluation system; Hitachi High-Tech Analysis Corp.). A CT15E centrifuge (Eppendorf Himac Technologies Co., Ltd., Ibaraki, Japan) was used for solid-phase extraction (SPE) spin column pretreatment of analyte-spiked liquid control serum I samples to provide HPLC test solutions according to the standardized procedures for each using the analyte manufacturer-recommended method for the LM1010 with the manufacturer-recommended method.

### Preparation of analyte calibration standard solutions

Standard solutions of acetaminophen, cefmetazole, cefazolin, amiodarone, and *N*-desethylamiodarone for calibration were prepared via accurate dilution of the respective calibration standard solutions to 6 to 10 concentrations. The specific concentrations and solvents for each analyte are summarized in Table 1. Three sets of calibration standard solutions were prepared for each analyte. The calibration concentration ranges for each analyte, as shown in Table 1, were selected based on their clinical relevance in actual clinical practice. For acetaminophen, the upper concentration was set at approximately 300 µg/mL (1,985 µmol/L) to cover the concentrations relevant for assessing acetaminophen intoxication. For amiodarone and N-desethylamiodarone, the calibration range was designed to cover the effective therapeutic concentration range of approximately 0.5–2 µg/mL, which corresponds to approximately 0.77–3.10 µmol/L for amiodarone and approximately 0.81–3.24 µmol/L for N-desethylamiodarone, respectively. This range corresponds to the concentrations relevant to therapeutic drug monitoring in clinical practice, and the lower end of the calibration range was set at 0.18 µg/mL as the limit of quantification (LOQ), corresponding to approximately 0.28 µmol/L for amiodarone and 0.30 µmol/L for N-desethylamiodarone. Cefazolin and cefmetazole are not currently included in routine therapeutic drug monitoring; nonetheless, their calibration ranges were determined after consultation with experts involved in the preparation of guidelines for antimicrobial drug monitoring to cover the concentrations considered clinically relevant for possible future applications.

**Table 1:** Concentration and solvent composition of calibration standard solutions of each analyte

Analyte	Concentration (µmol/L)	Solvent
Acetaminophen	4.20, 8.20, 16.3, 32.2, 64.2, 127, 249, 497, 981, 1985	10% (w/w) acetonitrile in water
Cefmetazole	1.68, 3.36, 6.69, 13.6, 26.8, 53.0, 106, 212	30% (w/w) acetonitrile in water
Cefazolin	1.81, 3.58, 7.10, 14.0, 27.9, 55.4, 110	50% (w/w) acetonitrile in water
*N*-desethylamiodarone	0.30, 0.58, 1.11, 2.14, 4.15, 8.10	Methanol
Amiodarone	0.28, 0.55, 1.06, 2.05, 3.97, 7.75	Methanol

### HPLC analysis of calibration standard solutions

Calibration standard solutions of each analyte were analyzed by HPLC with UV detection at 240 nm (amiodarone, *N*-desethylamiodarone), 245 nm (acetaminophen), 270 nm (cefazolin), and 275 nm (cefmetazole) using two dedicated mobile phases (mobile phases A and B; Hitachi High-Tech Analysis Corp.) and a LaChrome LM TypeA analytical column (Hitachi High-Tech Analysis Corp.). Standardized analytical conditions in accordance with the manufacturer-recommended method were employed. Mobile phase A consisted of an aqueous buffer, and mobile phase B consisted of acetonitrile. The chromatographic elution was performed using only these two mobile phases, which were mixed in a time-programmed gradient mode within the solvent delivery pump before delivery to the analytical column. Analyses of acetaminophen, cefmetazole, and cefazolin (including flushing and re-equilibration of the analytical column) were completed within 8 min.

**RMS calculations** The RMS of each analyte (relative to the non-analyte reference material) was calculated from the ratio of the slope of the calibration curve passed through the origin for each analyteand the non-analyte reference material based on the HPLC results [22]:

1 $$\:\begin{array}{c}RMS=\frac{\mathrm{S}\mathrm{l}\mathrm{o}\mathrm{p}\mathrm{e}\:\mathrm{o}\mathrm{f}\:\mathrm{c}\mathrm{a}\mathrm{l}\mathrm{i}\mathrm{b}\mathrm{r}\mathrm{a}\mathrm{t}\mathrm{i}\mathrm{o}\mathrm{n}\:\mathrm{e}\mathrm{q}\mathrm{u}\mathrm{a}\mathrm{t}\mathrm{i}\mathrm{o}\mathrm{n}\:\mathrm{f}\mathrm{o}\mathrm{r}\:\mathrm{a}\mathrm{n}\mathrm{a}\mathrm{l}\mathrm{y}\mathrm{t}\mathrm{e}}{\mathrm{S}\mathrm{l}\mathrm{o}\mathrm{p}\mathrm{e}\:\mathrm{o}\mathrm{f}\:\mathrm{c}\mathrm{a}\mathrm{l}\mathrm{i}\mathrm{b}\mathrm{r}\mathrm{a}\mathrm{t}\mathrm{i}\mathrm{o}\mathrm{n}\:\mathrm{c}\mathrm{u}\mathrm{r}\mathrm{v}\mathrm{e}\:\mathrm{f}\mathrm{o}\mathrm{r}\:\mathrm{n}\mathrm{o}\mathrm{n}\mathrm{a}\mathrm{n}\mathrm{a}\mathrm{l}\mathrm{y}\mathrm{t}\mathrm{e}\:\mathrm{r}\mathrm{e}\mathrm{f}\mathrm{e}\mathrm{r}\mathrm{e}\mathrm{n}\mathrm{c}\mathrm{e}\:\mathrm{m}\mathrm{a}\mathrm{t}\mathrm{e}\mathrm{r}\mathrm{i}\mathrm{a}\mathrm{l}}\end{array}$$

Carbamazepine and caffeine were used as non-analyte reference materials. Calibration equations generated under the previously reported standardized analytical conditions of the LM1010 evaluation system were used, as follows: $\:y\:=\:6614x$ for carbamazepine and $\:y\:=\:1400x$ for caffeine [22]. These calibration curves of the non-analyte reference materials were constructed at fixed detection wavelengths, namely 280 nm for carbamazepine and 270 nm for caffeine, which correspond to the absorption maxima (λ_max_) of these two compounds.

### Sample preparation for RMS analysis

The performance of the RMS method was evaluated using control serum samples spiked with each analyte at two or three concentrations for acetaminophen, cefmetazole, and cefazolin, and with both analytes at two concentration levels for *N*-desethylamiodarone and amiodarone. The spiked control serum samples were pretreated according to the manufacturer-recommended operating procedures for each analyte. Spiked control serum samples were initially filtered through a 0.45-µm syringe filter (Hitachi High-Tech Analysis Corp.) prior to processing. Filtered control serum samples were then subjected to SPE using spin columns optimized for each analyte, and the resulting filtrates were used as the test solutions. All centrifugation steps during the SPE process were carried out at 2,400 × *g* and 25 °C (Hitachi High-Tech Analysis Corp.). Three sets of test solutions were used for each analyte at different concentrations.

### Determination of analyte concentration

Analyte concentrations were determined using calibration curves for non-analyte reference materials (carbamazepine or caffeine) in conjunction with calculated RMS values. Calibration curves for the non-analyte reference materials were constructed using 8 concentrations over the range 0.9–84.6 µmol/L for carbamazepine and 10 concentrations over the range 1.0–515.0 µmol/L for caffeine, according to a previously reported method [22]. The concentration of each analyte in the respective control serum sample was calculated as follows:

2 $$\rm{{Concentration}}\:{{\mu g/mL}}{{ = C \times }}\frac{{{1}}}{{{{RMS}}}}{{ \times }}\frac{{{{M_{A}}}}}{{{{1}}000}}$$

where C represents the concentration of analyte in the test solution, as determined from the calibration curve of the non-analyte reference material (µmol/L), M_A_ represents the molecular weight of the analyte (g/mol), and RMS is given by Eq. (1).

For validation, the same test solutions were analyzed using the conventional method and analyte-specific calibration curves constructed from authentic reference standards. In this approach, the analyte concentration was calculated as follows:

3 $$\rm{{Concentration}}\ {{\mu g/mL}}{{ = C \times }}\frac{{{{M_{A}}}}}{{{{1}}000}}$$

Data are expressed as the mean and relative standard deviation (RSD).

## Results

### Linearity

**Fig. 2:**
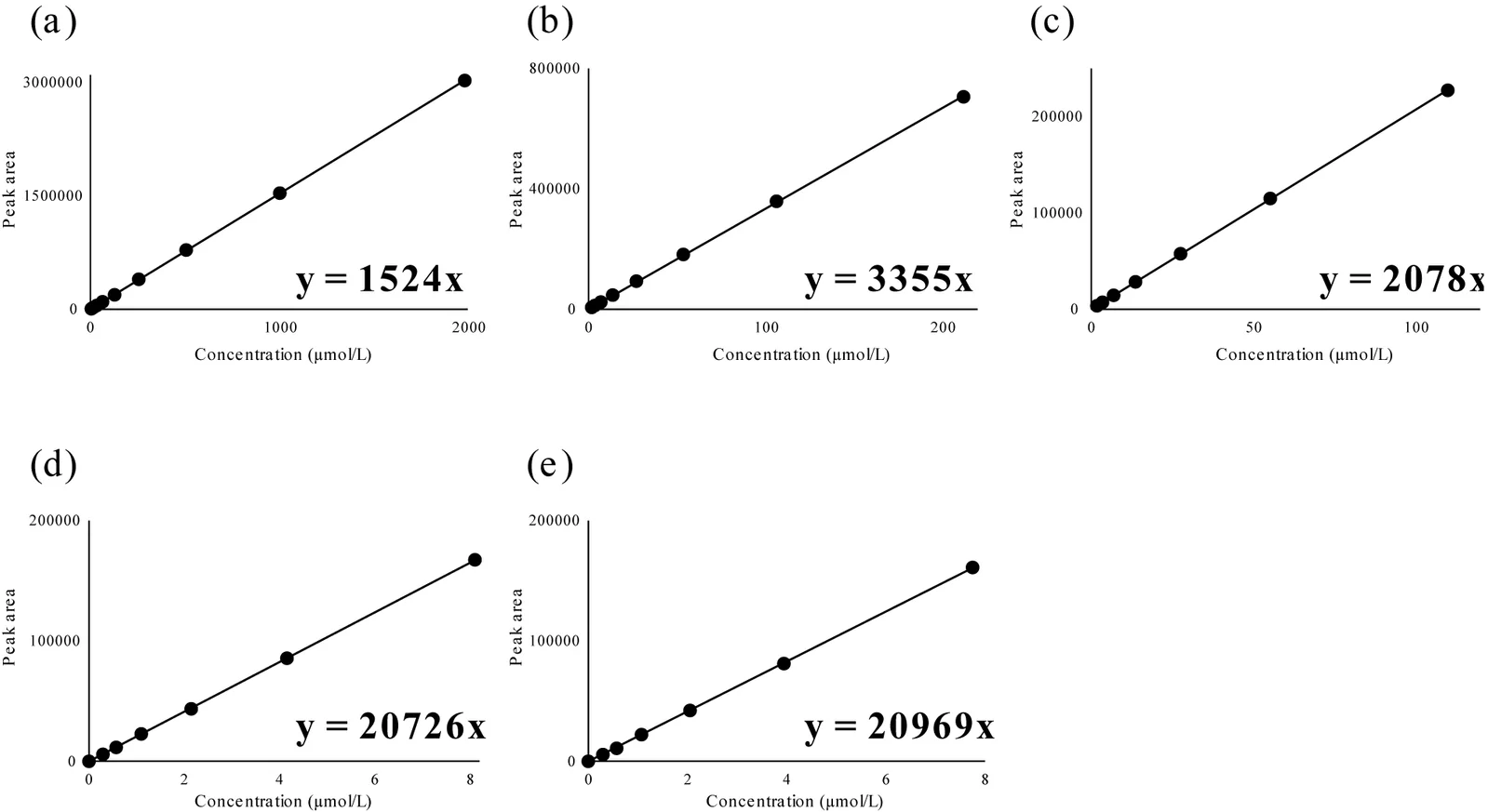
Calibration curves. Typical calibration curves are shown for: (**a**) acetaminophen, (**b**) cefmetazole, (**c**) cefazolin, (**d**) amiodarone, and (**e**) *N*-desethylamiodarone

**Fig. 3:**
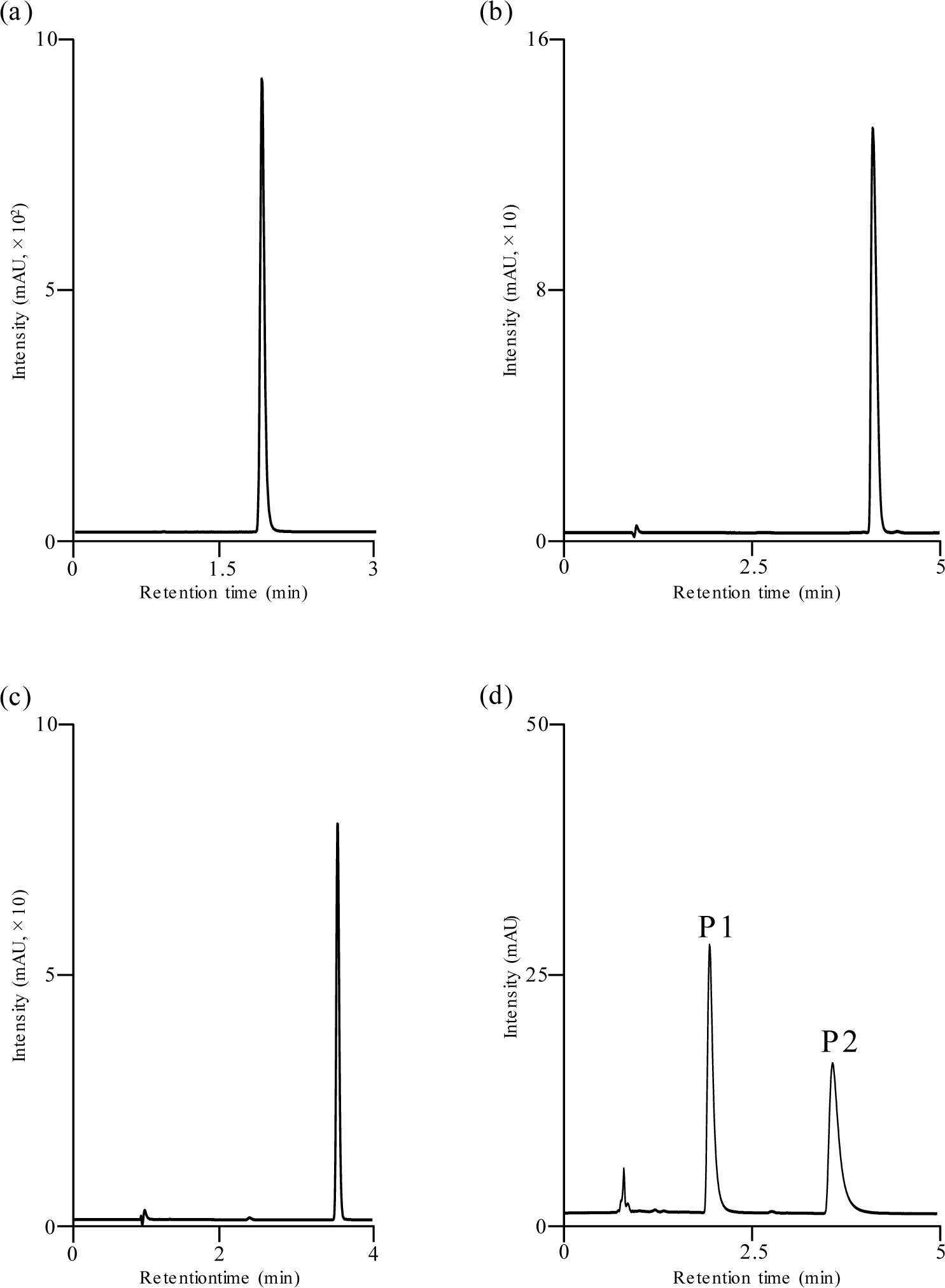
Analyte chromatograms. Typical high-performance liquid chromatography chromatograms are shown for: (**a**) acetaminophen, (**b**) cefmetazole, (**c**) cefazolin, and (**d**) *N*-desethylamiodarone (P1) and amiodarone (P2)

Linearity was evaluated using calibration standard solutions prepared at multiple concentrations (see Table 1). Calibration curves were constructed by plotting the exact concentration against the corresponding chromatographic peak area, passing through the origin (Figs. 2 and 3). The correlation coefficients of calibration curves for all analytes were in the range 0.9993–1.0000, and the RSDs of the calibration curve slopes ranged from 0.3% to 1.5% (Table 2). The limits of quantification for acetaminophen, cefmetazole, cefazolin, *N*-desethylamiodarone, and amiodarone were 1.1, 1.1, 1.8, 0.30, and 0.19 µmol/L, respectively, based on repeated analysis of standard solutions for each analyte prepared at a concentration assumed to be near the LOQ, which was defined as the concentration corresponding to ten times the standard deviation of the measured values.

**Table 2:** Regression data for each analyte

Analyte	Calibration equation	Relative standard deviation (%)	Correlation coefficient
Acetaminophen	$\:y\:=\:1524x$	0.8	0.9999–1.0000
Cefmetazole	$\:y\:=\:3355x$	0.5	0.9998–1.0000
Cefazolin	$\:y\:=\:2078x$	0.6	0.9999–1.0000
*N*-desethylamiodarone	$\:y\:=\:20726x$	1.5	0.9996–1.0000
Amiodarone	$\:y\:=\:20969x$	1.5	0.9993–1.0000

RMS values were calculated from slope ratios in which the analyte slope was obtained at the analyte-specific wavelength (240–275 nm) and the non-analyte reference material slope was obtained at its λ_max_-based fixed wavelength (carbamazepine, 280 nm; caffeine, 270 nm)

### Calculation of the RMS of five analytes against the non-analyte reference material

RMS values were calculated according to Eq. (1) with carbamazepine and caffeine as the non-analyte reference materials. The correlation coefficients and RSD values of the analyte calibration curves were > 0.999 and < 1.5%, respectively (Table 2). The average (*n* = 3) slope of the calibration curves for both the analytes and non-analyte reference materials were used in Eq. (1), yielding the RMS values shown in Table 3.

**Table 3:** Relative molar sensitivity of each analyte to the two non-analyte reference materials

	Non-analyte reference material	
	Carbamazepine	Caffeine
Acetaminophen	0.23	1.09
Cefmetazole	0.51	2.40
Cefazolin	0.31	1.49
*N*-desethylamiodarone	3.13	14.8
Amiodarone	3.17	15.0

### Comparison of the RMS-based and conventional HPLC methods

**Fig. 4:**
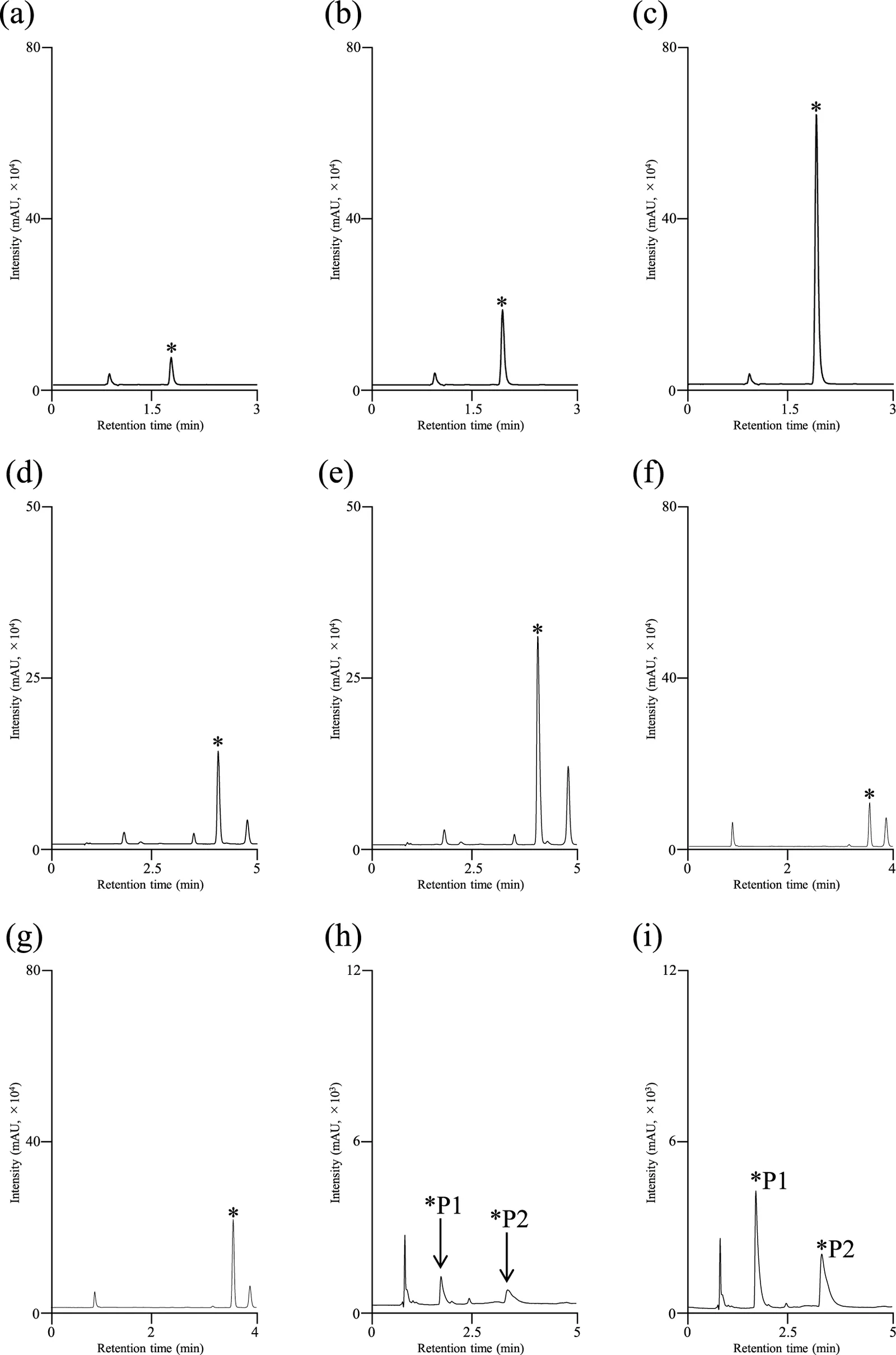
Spiked control serum sample chromatograms. Representative high-performance liquid chromatography chromatograms are shown for control serum samples spiked with: (**a**) Sample 1, (**b**) Sample 2, (**c**) Sample 3, (**d**) Sample 4, (**e**) Sample 5, (**f**) Sample 6, (**g**) Sample 7, (**h**) Sample 8, and (**i**) Sample 9 (Samples are described in Table 4). *: Analyte peak for quantification, *P1: *N*-desethylamiodarone, *P2: amiodarone

Analyte concentrations were measured using both the RMS-based and conventional absolute calibration HPLC methods (Table 4). Representative chromatograms of the analyte-spiked control serum samples are shown in Fig. 4. The concentrations determined by the two RMS-based methods showed no substantial differences from those obtained by the conventional method; the relative differences from the conventional method did not exceed ± 0.50% for either the RMS method using carbamazepine as the calibrant or the RMS method using caffeine as the calibrant, demonstrating close agreement between the RMS-based and conventional methods. The precision of both methods yielded RSD values of < 4.6% for replicate measurements. The absolute recovery of each analyte from the serum matrix under the present pretreatment conditions was as follows: acetaminophen, 109.7 ± 2.6% at 20 µg/mL and 102.7 ± 0.68% at 300 µg/mL; cefmetazole, 110.9 ± 1.3% at 2 µg/mL and 106.0 ± 2.9% at 100 µg/mL; cefazolin, 103.2 ± 6.1% at 2 µg/mL and 103.8 ± 2.3% at 100 µg/mL; N-desethylamiodarone, 101.7 ± 6.7% at 0.5 µg/mL and 100.0 ± 7.1% at 5 µg/mL; and amiodarone, 97.5 ± 2.9% at 0.5 µg/mL and 100.0 ± 8.0% at 5 µg/mL. These recovery data indicate that the analytes were satisfactorily recovered from the serum matrix, and, because the same pretreatment procedure was applied to both the RMS-based and conventional quantification methods, any effect of SPE recovery would have affected both methods under identical conditions.

**Table 4:** Comparison of analyte content in samples determined using two methods

	Sample designation	Spiked concentration (µg/mL)	RMS method				Conventional method	
			Calibrant: carbamazepine		Calibrant: caffeine			
			Concentration (µg/mL)	RSD	Concentration (µg/mL)	RSD	Concentration (µg/mL)	RSD
				(%)		(%)		(%)
Acetaminophen spiked	Sample 1	22	23.8	1.2	23.7	1.2	23.7	1.2
	Sample 2	53	56.2	2.1	56.0	2.1	56.0	2.1
	Sample 3	206	216	4.6	215	4.6	215	4.6
Cefmetazole spiked	Sample 4	21	20.8	3.1	20.8	3.1	20.9	3.1
	Sample 5	53	51.9	3.5	51.9	3.5	52.0	3.5
Cefazolin spiked	Sample 6	20	18.3	1.7	18.3	1.7	18.3	1.7
	Sample 7	50	45.7	2.1	45.7	2.1	45.5	2.1
*N*-desethylamiodarone spiked	Sample 8	0.55	0.467	1.8	0.468	1.8	0.469	1.8
	Sample 9	2.1	2.06	1.5	2.06	1.5	2.06	1.5
Amiodarone spiked	Sample 8	0.5	0.501	2.9	0.502	2.9	0.502	2.9
	Sample 9	1.9	1.99	2.3	1.99	2.3	2.00	2.3

## Discussion

The present study confirmed the analytical robustness of the RMS-based HPLC method across analytes with distinct physicochemical properties. The excellent linearity (*R*^*2*^ >0.999) and precision (RSD < 1.5%) of the calibration curves demonstrate the method’s stability for reliable RMS calculations. These performance metrics validate the core methodological premise that the detector response ratios remain constant under standardized chromatographic conditions.

The calculation of RMS values in this study was based on previously reported calibration equations for two non-analyte reference materials [22]. The calibration curves for both reference materials showed excellent linearity (*R²* >0.999) and precision (RSD < 0.6%), indicating that the calculated RMS values were derived from sufficiently reliable calibration curve slopes. In addition, the validity of calculating the RMS values under conditions in which the analyte and non-analyte reference material are monitored at different detection wavelengths has already been demonstrated in previous studies. In our previous report, nine pharmaceutical compounds were quantified across a wide range of detection wavelengths (220–295 nm) using the same two non-analyte reference materials, carbamazepine and caffeine, measured at their respective λ_max_-based fixed wavelengths, and the RMS-based results showed close agreement with those obtained using the conventional absolute calibration method [22]. A similar approach was also reported for acteoside and pedaliin, which were monitored at 340 nm while methyl 4-hydroxybenzoate was measured as the reference material at 254 nm, again yielding RMS-based results in close agreement with those of the conventional method [32]. These precedents indicate that once the RMS value is empirically determined under fixed chromatographic and spectrophotometric conditions, differences in the detection wavelength between the analyte and reference material do not compromise the accuracy of RMS-based quantification. Therefore, the wavelength combinations employed in this study are considered appropriate for RMS calculation without introducing an additional cross-wavelength correction factor.

The accuracy of the proposed RMS method was substantiated by the agreement between the RMS-based and conventional absolute calibration methods for all spiked samples. The consistent performance across five analytes with distinct physicochemical properties demonstrates the RMS-based method’s robustness and versatility and further indicates the absence of systematic bias across the different concentrations and chemical structures examined. This consistency is particularly significant given the range of molecular weights and chemical characteristics represented by the selected compounds, which ranged from small hydrophilic molecules (e.g., acetaminophen) to large lipophilic structures (e.g., amiodarone).

Although these findings support the robustness of the RMS-based approach under the present conditions, the reported RMS values should be interpreted in the context of the analytical instrument used in this study. The RMS values were obtained using the LM1010 evaluation system, in which the analytical parameters, dedicated column, and dedicated mobile phases were optimized and pre-installed by the manufacturer, and daily QC measurements with predefined acceptance criteria, together with scheduled maintenance, ensured stable and reproducible chromatographic performance. Accordingly, the reported RMS values should be regarded as instrument-specific parameters rather than universally transferable parameters. Their direct transfer to HPLC systems from other manufacturers or to non-dedicated columns cannot be assumed without verification, as detector response and chromatographic conditions may differ.

Another limitation of the present study is that method validation was conducted using only spiked control serum, and formal cross-validation using patient samples was not performed. For the analytes examined here, patient samples were already analyzed in clinical practice using the conventional method, with the same pretreatment procedure and LC analytical conditions as those applied in this study, and no obvious interference attributable to concomitant medications or endogenous patient-derived peaks has been recognized in routine practice. Nevertheless, the possible influence of concomitant medications and endogenous matrix components in real clinical samples cannot be completely excluded based on the present data alone. Future comparative validation of the RMS-based method against the existing method using patient samples will therefore be an important direction for subsequent work on the analytes investigated in this study. The comparable accuracy and precision of the RMS-based and conventional methods shows that use of the RMS-based method eliminates the need for analyte-specific CRMs while maintaining analytic reliability, thereby reducing the complexity and costs of procurement. A key advantage of this approach is the strategic use of carbamazepine and caffeine as non-analyte reference materials. These CRMs are chemically stable and available with established absolute purity traceable to the International System of Units [15, 22]. Eliminating the need for analyte-specific CRMs is particularly beneficial for analyses of beta-lactam antibiotics (e.g., cefmetazole and cefazolin), which are unstable even under refrigerated conditions [33, 34]. In this study, to minimize any potential degradation of these unstable analytes, spiked serum samples were aliquoted and stored at − 30 °C and thawed at 25 °C immediately before analysis to reduce the number of freeze–thaw cycles. Under these storage conditions, the two beta‑lactam antibiotics remained stable at − 30 °C for more than 210 days, with no appreciable loss observed. Furthermore, the recovery data for cefmetazole and cefazolin, together with the quantified values obtained from the spiked serum samples (Table 4), support the hypothesis that no appreciable degradation of these analytes occurred during the thawing process under the present experimental conditions. The proposed method also mitigates the risk of CRM decomposition during storage, in turn reducing the frequency and logistical burden of repurchasing expensive reference materials. In addition, the total analysis time per sample of the optimized chromatographic method is only 8 min for beta-lactam antibiotics (e.g., cefmetazole and cefazolin). This rapid turnaround time enables expedited result reporting to clinicians, supporting timely therapeutic decision-making and dose adjustments [8]. These findings demonstrate the potential of the RMS-based HPLC method for routine therapeutic monitoring of multiple drugs in clinical laboratories.

## Conclusions

This study developed and validated an RMS-based HPLC analytical method for the quantification of acetaminophen, cefmetazole, cefazolin, amiodarone, and *N*-desethylamiodarone. The proposed RMS-based method exhibited good accuracy, precision, and linearity within the assessed concentration ranges, with quantitative results demonstrating excellent concordance with conventional absolute calibration–based techniques for all samples. The optimized chromatographic method achieves rapid analysis, which enables near–real-time result reporting to support dosage optimization and therapeutic management. Thus, the proposed RMS-based method provides a promising practical approach for rapid routine clinical quantitative analyses. The method offers several advantages, such as greater cost-effectiveness and shorter turnaround time compared with conventional methods. Future development should focus on expanding this analytical platform to encompass a broader range of TDM-relevant pharmaceuticals and on integrating the method into automated hospital laboratory systems. Such advances would support precision medicine initiatives and enhance patient safety through the optimization of dosing and minimization of adverse drug reactions.

## Data Availability

No datasets were generated or analysed during the current study.

## Abbreviations

TDM: Therapeutic drug monitoring
HPLC: High-performance liquid chromatography
RMS: Relative molar sensitivity
CRM: Certified reference material
SI: The International system of units
SPE: Solid-phase extraction
RSD: Relative standard deviation
UV: Ultraviolet
